# Construction Notes

**Published:** 1895-06-08

**Authors:** 


### CONSTRUCTION NOTES.

**New Operating Theatre at Dundee Infirmary.**

The operating theatre which it was resolved should be added to the Dundee Royal Infirmary has been commenced, and it is hoped the building will be completed by the end of the year. The new buildings are to be erected at the N.W. of the present hospital and connected by a corridor which opens into the anæsthetic-room. Communicating with this latter is the surgeons'-room, next to which is the sterilising and instrument-room. The theatre itself will be a handsome room 30 ft. by 25. At the north end accommodation is provided for 40 students. The floor will be of the latest and most approved Taratza. It will be lighted by electricity. The estimated cost of this much-needed addition to the hospital will be £1,500. Messrs. Robert Lang are the builders of the masonry.

**New Small-pox Hospital at Birmingham.**

This hospital is situated on the east side of the city, about 500 yards within the city boundary. The site comprises nearly twenty-four acres. The entrance lodge, a red-brick building relieved with stone, is immediately to the right of the large entrance gates. The receiving ward comprises a single room, with a bath-room leading therefrom, and a small enclosed yard and offices. The isolation pavilion is situated in a bye-road; it is arranged for males and females, and consists of a number of small wards with one, two, or three beds in each as required, and the access to each ward is from the open air, but under a covered glass verandah. Special bathing and other sanitary arrangements are made, all securing perfect sanitation. The official administrative block is sub-divided into three sections—the central, containing the medical quarters and matron's apartments; the left hand, containing the steward's apartments, stores, mess-room, kitchen, and kitchen yard; and the right hand containing the servants' accommodation and recreation room, the matron's stores, and official laundry. In this block is placed the telephone apparatus, by means of which communication can be had with all the various blocks. The pavilions for the reception of patients are four in number. Each of these accommodates 24 beds and consists of two wards 72 ft. by 30 ft., with an allowance of air-space to each patient of over 2,000 cubic ft. At the end of the wards are the bath-rooms, &c., fitted up with the latest sanitary improvements. The wards are heated by open-fire down-draught stoves. The ventilation is ample and controllable, being a natural system of extraction and diffusion. Oak blocks polished in the ordinary way with hard paraffin form a substantial flooring. The lighting is from the ceiling of the wards, the products of combustion being kept entirely out of the wards. The servants' quarters and cottages for the outdoor officials contain kitchens, general sitting-rooms, &c., and a small bed-room, thoroughly heated, lighted, &c., is provided for each servant. At the extreme rear of the centre of the hospital site is the laundry and disinfecting yard, with incinerator, perfect isolation being preserved all through. The steam disinfector is of the Washington-Syms type. The discharging ward is a small two-storeyed building with necessary dressing and bath rooms. The buildings have been erected by Mr. W. Robinson, of Birmingham, and the whole of the work has been carried out under the supervision and direction of the architect, Mr. W. H. Ward.

